# Transverse facial cleft repair: Preventing pigmentation around the postoperative scar by excising the pigmented white lip at the cleft margin—A retrospective case series of Japanese patients^✰^

**DOI:** 10.1016/j.jpra.2026.02.001

**Published:** 2026-02-10

**Authors:** Motomu Suito, Ikkei Takashimizu, Fumio Nagai, Yuki Hoshino, Masahiko Noguchi, Shunsuke Yuzuriha

**Affiliations:** a Department of Plastic and Reconstructive Surgery, Shinshu University School of Medicine, Matsumoto, Nagano 390-8621, Japan; b Department of Plastic Surgery, Aizawa Hospital, 2-5-1 Honjou, Matsumoto, Nagano 390-8510, Japan; c Department of Plastic Surgery, Nagano Matsushiro General Hospital, 183 Matsushiro, Matsushiro, Nagano 381-1231, Japan; d Department of Plastic Surgery, Nagano Children’s Hospital, Azumino, Nagano 399-8288, Japan

**Keywords:** Transverse facial cleft, Tessier number 7 cleft, Lateral facial cleft, Macrostomia, Pigmentation, Scar

## Abstract

Numerous transverse facial cleft repair techniques and designs have been described; however, a complication of surgical reconstruction is scar formation with brown discoloration. This study investigated the histological basis of the darker white lip at the cleft margin, its role in postoperative brown discoloration, and the potential esthetic benefits of extending the excision.

In this retrospective analysis, patients with transverse facial cleft were divided into limited and extended excision groups. Histological assessment of melanin pigment was performed using Fontana–Masson staining, and postoperative buccal scars were evaluated from photographs using the Stony Brook Scar Evaluation Scale. The Mann–Whitney *U* test was used for statistical analysis.

We divided 16 Japanese patients (4 boys and 12 girls) into limited (*n* = 9) and extended excision groups (*n* = 7). The cleft length was 8.6 ± 2.1 and 8.9 ± 3.3 mm in the limited and extended excision groups, respectively, with unilateral/bilateral laterality being 9/0 and 6/1, respectively. Initial surgeries for both groups were performed at 11.4 ± 8.4 and 8.3 ± 7.7 months, and scar evaluations were based on photographs obtained at 47.8 ± 13.5 and 27.1 ± 14.2 months, respectively. Histology showed increased melanin in the darker area of the white lip at the cleft margin, with markedly reduced pigment density in the normal-toned area. The extended excision group had less colored scar than the limited excision group (*p* < 0.05).

Incorporating pericleft pigmentation excision into surgical design may reduce postoperative periscar hyperpigmentation and improve esthetic outcomes for several years.

## Introduction

Transverse facial cleft, also known as Tessier number 7 cleft, lateral facial cleft, or macrostomia, is a phenotypic manifestation of anomalies in the first and second branchial arches.^1^^,^^2^ It is not an isolated deformity but is often accompanied by soft- and hard-tissue anomalies resulting from failed fusion of the maxillary and mandibular processes, with varying degrees of facial asymmetry.^3^^,^^4^ Common associated anomalies include underdevelopment of the external and/or middle ear, facial and masticatory muscles, palatal muscles, tongue, parotid gland, temporal bone, maxilla, zygoma, and mandible, as well as accessory tragus, skin tags, branchial cleft sinus, seventh cranial nerve involvement, maxillary duplication, and accessory maxilla.^2^^,^^5^^,^^6^ Additionally, in a series of 102 patients with first and second branchial arch syndrome, the most frequently observed congenital malformations included vertebral/rib malformations (11%), ocular anomalies (10%), and cleft lip/palate (7%).^2^ Transverse facial cleft is frequently associated with hemifacial microsomia.^2^^,^^7^^,^^8^ It has also been reported, although less commonly, in Goldenhar syndrome (a variant of hemifacial microsomia) and in Treacher Collins syndrome.^8^^,^^9^ Most clefts are unilateral, and although some might extend laterally toward the tragus, they typically do not reach the anterior border of the masseter muscle, which consequently limits the cleft length to approximately 1–2 cm.^2^^,^^3^^,^^10^

Various surgical techniques have been described for repairing transverse facial clefts to achieve a natural lip appearance, restore normal mouth function, and minimize visible scarring. Z-plasty,^7^ W-plasty,^11^ and straight-line closure^12^ are the most commonly used skin closure techniques. However, a brown-colored area around the postoperative scar in the buccal region has been observed in some postoperative photographs in the published literature.^13^^,^^14^ We also encountered such cases despite appropriate postoperative wound management. These observations suggest that brown discoloration around the postoperative scar may be related to preoperative factors rather than postoperative factors. Accordingly, we focused on the macroscopically darker area of the white lip around the cleft, which was originally present at the cleft margin, and hypothesized that the melanin pigment left behind at the cleft margin during commissuroplasty may have caused the brown-colored area around the postoperative scar. Based on this hypothesis, our surgical approach was modified. At our institution, cleft tissue was excised along the vermilion–cutaneous junction until 2006. Since 2007, the macroscopically darker area of the white lip surrounding the cleft has been excised during commissuroplasty.

This study aimed to investigate the underlying cause of the brown-colored area around the postoperative buccal scar and evaluate whether extensive skin excision at the cleft margin, including the macroscopically darker area of the white lip, results in less conspicuous postoperative scarring.

## Patients and methods

### Study design and patients

This retrospective observational study included Japanese patients with transverse facial clefts who underwent surgical repair at Shinshu University Hospital or Nagano Children’s Hospital between 1989 and 2021 and had complete clinical records and photographs. Patients were divided into two groups according to the extent of tissue excision around the cleft margin during commissuroplasty: the limited excision group, which underwent excision along the vermilion–cutaneous junction, and the extended excision group, which underwent additional excision including the macroscopically darker area of the white lip at the cleft margin. The cleft tissue excised in the extended excision group was examined histologically to determine why a macroscopically darker area of the white lip developed at the cleft margin. Postoperative scar appearance was evaluated using blinded image assessment panels, and the results were statistically analyzed. This study was approved by the Ethics Committee of Shinshu University School of Medicine (approval number: 5846).

### Surgical procedures and postoperative wound management

The surgical design for unilateral transverse facial cleft repair was as follows: First, the following anatomic landmarks were marked on the noncleft side: upper and lower lip midline (*labialis superioris* [*ls*] and *labialis inferioris* [*li*], respectively), both sides of the cupid’s bows (*crista philtra inferioris* [*cphi*]), and *commissure* (*ch*). After measuring the distances from the *cphi* to the *ch* and from the *li* to the *ch* on the noncleft side, the same distances were marked on the upper and lower lip on the cleft side. The skin excision area was outlined along the margin of the vermilion–cutaneous junction in the limited excision group, whereas it was designed to include the macroscopically darker area of the white lip around the cleft margin in the extended excision group. For skin closure, a Z-plasty–based design was used in both groups. After excising the excess cleft tissue, the laterally displaced *orbicularis oris* muscle was corrected, and the upper and lower muscle bundles were sutured together using 5–0 polydioxanone. The intraoral mucosa was closed using a 5–0 braided absorbable suture (Coated Vicryl®, Ethicon Inc., Somerville, NJ, USA), and the subcutaneous tissue was closed using a 6–0 monofilament polydioxanone suture (PDS®, Ethicon Inc.). The skin was closed using a 7–0 monofilament nonabsorbable suture (Prolene®, Ethicon Inc.) without tightening the suture, which was removed on postoperative day 5, after which adhesive tape was applied for light shielding.

### Histological investigation of excised cleft tissue

The cleft tissue excised from a patient in the extended excision group was evaluated histologically using Fontana–Masson staining to assess the quantity and distribution of melanin pigment. The extent of melanin staining was quantified using the Vectra® 3.0 System (PerkinElmer Inc., Waltham, MA, USA) to calculate the percentage of Fontana–Masson stain–positive cells within evenly sized unit areas across the mucosa, vermilion, and white lip.

### Evaluation and statistical analysis of postoperative scar features

The appearance of postoperative buccal scars was evaluated on the frontal photographs by three groups of blinded evaluators using the Stony Brook Scar Evaluation Scale (SBSES) (Table 1).^15^ The SBSES is a validated tool used to assess the appearance of surgical scars and includes five dichotomous categories: width, height, color, suture marks, and overall appearance. Each category is scored either 0 or 1. The total score ranges from 0 to 5, with higher scores indicating better scar quality and a more aesthetically favorable scar appearance.

**Table 1:** Stony Brook Scar Evaluation Scale.

Scar category	Number of points
**Width**	
>2 mm	0
≤2 mm	1
**Height**	
Elevated or depressed in relation to surrounding skin	0
Flat	1
**Color**	
Darker than surrounding skin (red, purple, brown, or black)	0
Same color or lighter than surrounding skin	1
**Hatch marks or suture marks**	
Present	0
Absent	1
**Overall appearance**	
Poor	0
Good	1

**Total score** (sum of the individual scores)	0–5 (from worst to best)

A total of 30 individuals were randomly selected as the image assessment panel, including 10 plastic surgeons (Group A), 10 junior residents (physicians with <2 years of experience since obtaining their medical license and without specialization in any specific medical field) (Group B), and 10 laypeople (Group C). The evaluators were blinded to the clinical data of the patients. The appearance of scars was evaluated by reviewing frontal photographs of each patient taken at 0–5 years of age and >6 months after surgery. None of the patients underwent surgical revision or any laser-based intervention between the initial surgery and the photographic evaluation.

The SBSES scores for the limited and extended excision groups, as assessed by each evaluator group (Groups A, B, and C) were aggregated. The six scores of the SBSES categories (five scar categories and the total score) evaluated by each evaluator group were compared and analyzed between the patient groups using the Mann–Whitney *U* test in R (version 4.3.1). A *p*-value of < 0.05 was considered significant.

## Results

### Patient characteristics

A total of 18 Japanese patients (5 boys and 13 girls) with transverse facial clefts who underwent surgical repair between 1989 and 2021 were enrolled in this study. Two patients (one boy and one girl) were excluded because of the lack of appropriate postoperative photographs. Finally, 16 patients (4 boys and 12 girls) were included in the analysis: 9 patients (4 boys and 5 girls) were assigned to the limited excision group and 7 patients (all girls) to the extended excision group. Table 2 summarizes their clinical data. All patients had hemifacial microsomia and no history of conditions associated with pigmentation around the oral commissures. Unilateral clefts were observed in 15 patients (4 right-sided and 11 left-sided), and 1 patient had a bilateral cleft that was repaired only on the right side because the cleft on the left side was too subtle to be recognized. The cleft length was 8.6 ± 2.1 and 8.9 ± 3.3 mm in the limited and extended excision groups, respectively. Initial surgery was performed at 11.4 ± 8.4 months in the limited excision group and 8.3 ± 7.7 months in the extended excision group, and postoperative scars were evaluated using photographs taken at 47.8 ± 13.5 and 27.1 ± 14.2 months, respectively. The most frequent associated anomalies were accessory tragus (13/16), microtia (5/16), and mandibular hypoplasia (5/16).

**Table 2:** Patient characteristics.

Characteristics	Limited excision group	Extended excision group	Total
**Number of the patients (boys/girls)**	9 (4/5)	7 (0/7)	16 (4/12)
**Laterality**			
Right/left/bilateral	2/7/0	2/4/1	16 (4/11/1)
**Cleft length (mm)**			
Mean ± SD	8.6 ± 2.1	8.9 ± 3.3	8.7 ± 2.8
Range	4–11	5–15	4–15
**Associated anomalies (unilateral/bilateral)**			
Accessory tragus	7 (5/2)	6 (4/2)	13 (9/4)
Microtia	2 (1/1)	3 (2/1)	5 (3/2)
Mandibular hypoplasia	1 (1/0)	4 (4/0)	5 (5/0)
Cheek tags	1 (1/0)	2 (2/0)	3 (3/0)
External auditory canal stenosis	2 (1/1)	1 (1/0)	3 (2/1)
External auditory canal atresia	1 (1/0)	0	1 (1/0)
Mandibular ramus defect	0	1 (1/0)	1 (1/0)
Ankyloglossia	1	0	1
Cleft palate	0	1 (1/0)	1 (1/0)
Cleft lip and palate	0	1 (1/0)	1 (1/0)
Cleft lobule	0	1 (1/0)	1 (1/0)
**Age at the time of surgery (months)**			
Mean ± SD	11.4 ± 8.4	8.3 ± 7.7	9.9 ± 8.2
Range	3–24	3–26	3–26
**Age at the time of evaluation (months)**			
Mean ± SD	47.8 ± 13.5	27.1 ± 14.2	38.8 ± 17.2
Range	27–65	10–48	10–65
**Scar age (months)**			
Mean ± SD	31.7 ± 16.0	18.9 ± 12.2	25.3 ± 15.1
Range	6–57	7–41	6–57
**Age at the last consultation (months)**			
Mean ± SD	92.8 ± 60.0	63 ± 42.6	79.8 ± 55.1
Range	30–209	12–149	12–209

SD: standard deviation.

The postoperative course was favorable in both groups. The repaired commissures could move naturally, and their three-dimensional shape was normal even during mouth opening. The continuity of the vermilion border at the new commissure was satisfactory.

### Histological findings of excised cleft tissue

**Figure 1:**
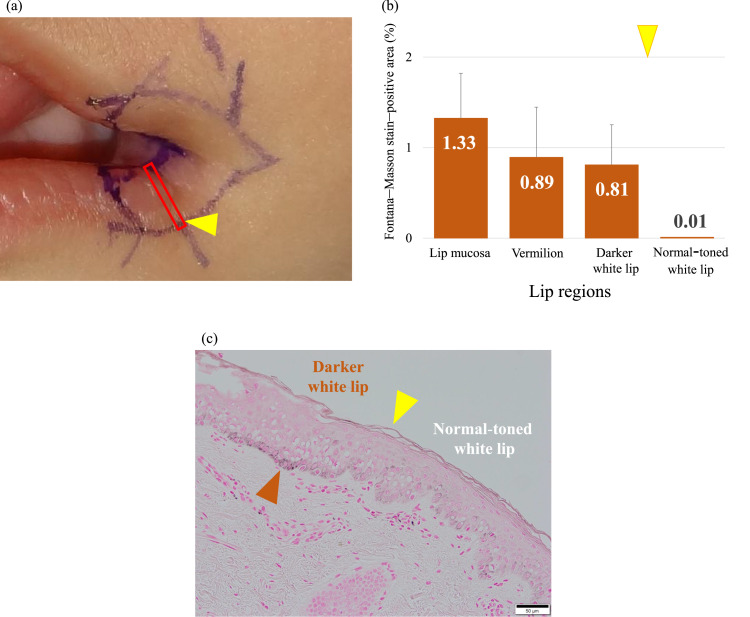
Histological examination of the cleft tissue. Yellow triangles indicate the border between the macroscopically darker area and the normal-toned area of the white lip. (a) Pathological observation site (red rectangle). (b) The graph shows the mean area percentage of Fontana–Masson stain-positive cells per unit area of the cleft tissue. In the macroscopically darker area of the white lip, a Fontana–Masson stain-positive area was observed (0.81%). Conversely, the normal-toned area of the white lip showed a markedly reduced Fontana–Masson stain-positive area (0.01%). (c) Fontana–Masson staining of the white lip (scale bar = 50 μm). Fontana–Masson stain-positive cells were observed at the basal layer of the lip epidermis (brown triangle) and were barely present outside the yellow triangle (right side of the triangle, macroscopically normal-toned area of the white lip).

Histological evaluation of the cleft tissue from a patient in the extended excision group revealed a predominant presence of Fontana–Masson stain–positive melanin granules in the basal layer of the lip mucosa, vermilion, and white lip epithelium (Figure 1a). The mean area percentages of melanin-positive regions were 1.33% ± 0.50% for the lip mucosa, 0.89% ± 0.55% for the vermilion, 0.81% ± 0.44% for the macroscopically darker area of the white lip, and 0.01% for the macroscopically normal-toned area at the outermost region of the white lip (Figure 1b). Melanin-positive regions identified by Fontana–Masson staining were predominantly observed in areas that corresponded to the macroscopically darker area noted intraoperatively and were markedly reduced in the outermost part of the white lip (Figure 1c).

### Statistical comparison of SBSES scores between the limited and extended excision groups

Table 3 shows the SBSES category scores and statistical analysis results comparing the limited and extended excision groups for each evaluator group. In all evaluator groups, scores for “color,” “overall appearance,” and “total score” were significantly higher in the extended excision group than in the limited excision group, with *p* < 0.05 across the categories.

**Table 3:** Statistical analysis of SBSES evaluation.

SBSES category	Evaluator group (*n* = 10 each)	Points awarded, median (interquartile range)		*U*-statistic	*p-*values	ICC (2.1)
		Limited excision group (*n* = 9)	Extended excision group (*n* = 7)			
**Width**	A	8 (6–9)	10 (9.5–10)	16	0.086	0.39
(out of 10)	B	8 (5–8)	10 (8.5–10)	11.5	0.030	0.21
	C	4 (1–5)	9 (7.5–9.5)	7	0.009	0.35
**Height**	A	6 (4–9)	8 (6.5–9)	21	0.262	0.26
(out of 10)	B	7 (5–9)	9 (8–9)	21.5	0.281	0.27
	C	6 (5–9)	9 (8–9.5)	17.5	0.131	0.45
**Color**	A	3 (0–5)	9 (8.5–10)	9.5	0.018	0.60
(out of 10)	B	2 (0–5)	9 (6.5–9)	11	0.026	0.54
	C	2 (1–6)	8 (6–10)	12	0.037	0.51
**Marks**	A	5 (2–6)	8 (7–9.5)	4.5	0.004	0.22
(out of 10)	B	9 (8–9)	9 (8.5–9.5)	24.5	0.434	0.06
	C	6 (5–7)	8 (7.5–9)	9.5	0.019	0.07
**Overall appearance**	A	5 (1–8)	9 (8–9.5)	11	0.028	0.44
(out of 10)	B	7 (4–9)	10 (8–10)	12	0.036	0.23
	C	4 (1–7)	9 (6–10)	11	0.029	0.45

**Total score**	A	27 (18–35)	44 (41.5–46)	6.5	0.008	0.61
(out of 50)	B	35 (25–38)	46 (39.5–47.5)	10.5	0.026	0.48
	C	24 (15–30)	45 (32.5–45)	8.5	0.014	0.54

ICC: intraclass correlation coefficient

## Case description

### Limited excision group

**Figure 2:**
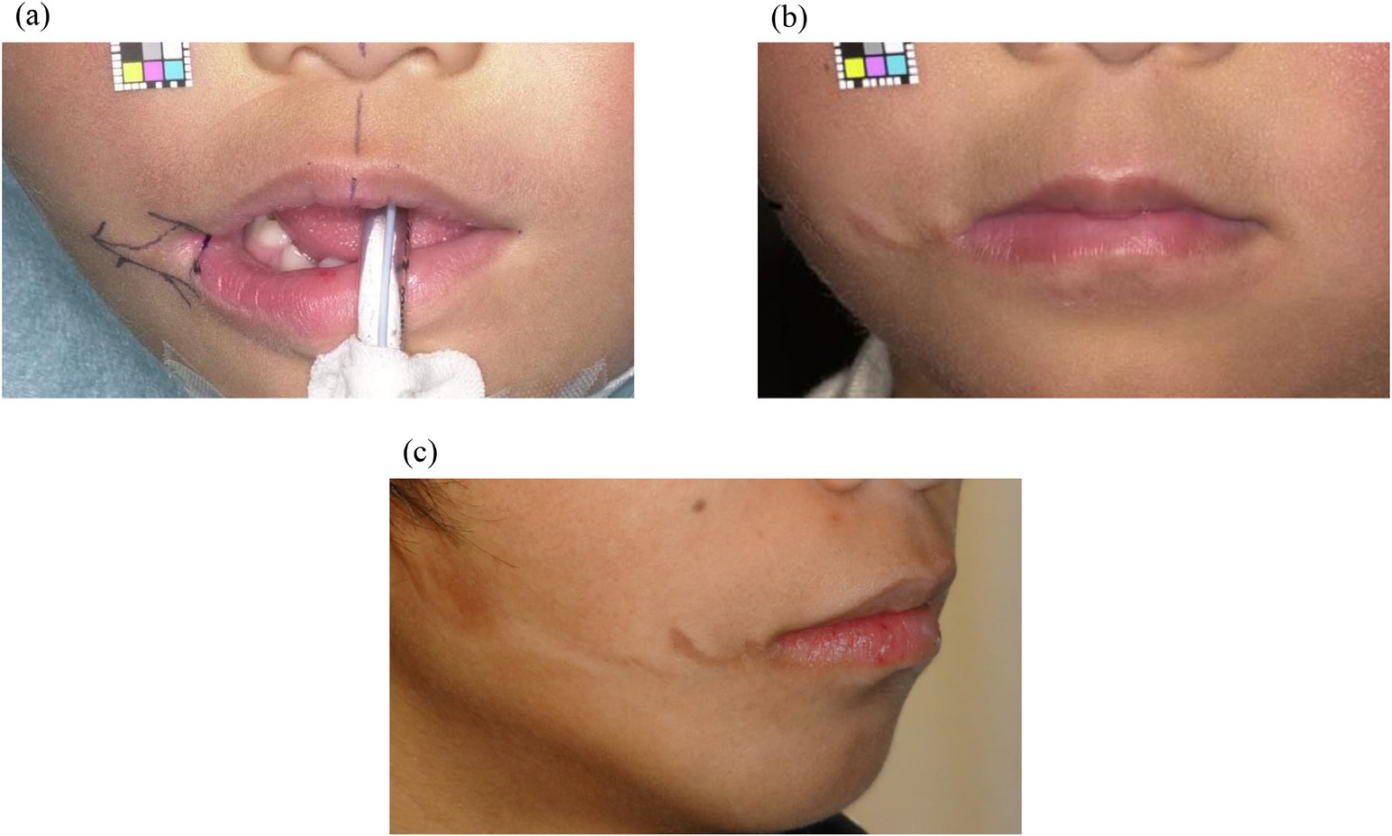
Limited excision group. (a) Surgical design for a 2-year-old boy. The skin excision area of the cleft was marked along with the vermilion border. (b) A brown-colored area was observed around the postoperative scar 25 months after the operation. (c) The color tone of the brown-colored area around the postoperative scar increased 15 years after the operation.

The patient was a 24-month-old boy with a right transverse facial cleft who underwent surgery. During the procedure, the cleft skin excision area was marked along the vermilion–cutaneous junction (Figure 2a). A brown-colored area was observed around the postoperative buccal scar 25 months after the operation (Figure 2b). At 17 years of age, an increase in the color intensity of the brown-colored area was observed (Figure 2c), and the patient underwent excision surgery due to the conspicuous scar.

### Extended excision group

**Figure 3:**
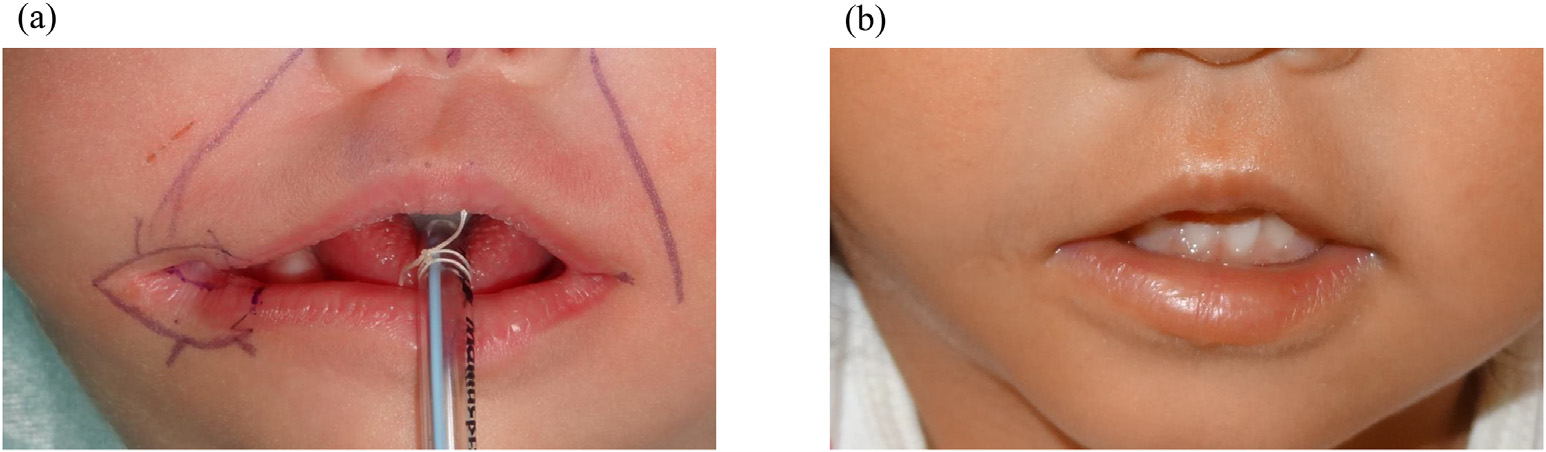
Extended excision group. (a) Surgical design for a 3-month-old girl. The skin excision area was marked with the macroscopically darker area of the white lip around the cleft. (b) No brown-colored area was observed around the postoperative scar 33 months after the operation.

The patient was a 3-month-old girl with a right transverse facial cleft who underwent surgery. During the procedure, the skin excision was designed to include the entire macroscopically darker area of the white lip adjacent to the cleft margin (Figure 3a). No brown-colored region was observed around the postoperative scar, and favorable esthetic outcomes were achieved (Figure 3b).

## Discussion

The standard surgical procedure for transverse facial cleft repair involves removing excess cleft tissue, correcting *orbicularis oris* muscle abnormalities, and finally closing the skin. Our examination of previously reported photographs of transverse facial cleft repair^13^^,^^14^^,^^16^ revealed that greater excision of the skin around the cleft, specifically, the excision of the macroscopically darker area of the white lip at the cleft margin, was associated with a reduced brown-colored area around the postoperative buccal scars, irrespective of the skin closure technique. Comparison of the limited excision design, which involves excising the cleft tissue along the vermilion–cutaneous junction,^13^^,^^14^ and the extended excision design, which involves excision of the macroscopically darker area of the white lip at the cleft margin,^16^ showed that the extended excision resulted in a less brown-colored area around the postoperative scars. Therefore, we focused on the macroscopically darker area of the white lip and hypothesized that this darker area was attributable to melanin pigmentation and that if this melanin-rich region was left unexcised during surgery, the residual pigmentation might contribute to the development of brown-colored areas around the postoperative scar.

A macroscopically darker area is commonly observed at the white lip surrounding the oral commissure, even among healthy individuals. This finding is observed irrespective of age and sex and varies among individuals. Similarly, a macroscopically darker area of the white lip at the cleft margin is observed in patients with a transverse facial cleft. Histological examination of the excised cleft tissue using Fontana–Masson staining revealed positively stained granular deposits within the cytoplasm of basal layer cells, a morphological feature characteristic of melanin pigment derived from melanocytes (Figure 1c). Additionally, a broad distribution of melanin pigment was observed, corresponding to the macroscopically darker area of the white lip, at the cleft margin. In contrast, the amount of melanin pigment decreased sharply in the outermost region, which appeared to be a macroscopically normal-toned area. Skin pigmentation and its intensity are primarily determined by the amount of melanin pigment produced by melanocytes.^17^^,^^18^ Based on these findings, the macroscopically darker area at the cleft margin is attributed to melanin pigmentation.

Postoperative scar features were evaluated by three evaluator groups using frontal photographs. Among the various scar assessment tools, including the Manchester Scar Scale^19^ and the Patient and Observer Scar Assessment Scale,^20^ the SBSES was used because (1) it contained “color” in its evaluation categories, (2) each scar category was evaluated using a 2-point scale, which is simpler than a ≥ 4-point scale that is considered difficult for evaluators to score according to a certain standard, and (3) it is a scar assessment system based only on the evaluator. Patient scar assessments were not performed because this was a retrospective study involving infants, from whom such assessments could not be obtained. For the evaluation, photographs taken at 0–5 years of age and >6 months postoperatively were used. These conditions were implemented to minimize the influence of sexual hormone–related pigmentation changes and allow resolution of minor postoperative inflammatory reactions at the surgical scars. With these criteria applied, analysis of the postoperative photographs showed that all scars had matured. Although no pigmentation was observed within the scars themselves, changes in skin tone were evident in the surrounding areas.

Postoperative scar evaluation using the SBSES revealed that the extended excision group had less colored and more aesthetically favorable scars than the limited excision group. Complete excision of the pigmented white lip at the cleft margin prevented the brown-colored area around the postoperative scar and resulted in a favorable scar appearance (Figure 3). Conversely, with limited excision of the pigmented white lip at the cleft margin, a brown-colored area was observed around the postoperative buccal scar (Figure 2). Furthermore, the color intensity of the brown-colored area remained persistent during subsequent growth and increased during puberty. Various factors that can contribute to increased pigmentation include sun exposure, inflammation, skin phototype, race, sex hormones, aging, endocrine disorders, and certain medications.^21^^,^^22^ Sun exposure is a common cause of hyperpigmentation, as melanin functions as a protective response against ultraviolet (UV) radiation.^22^ Postinflammatory hyperpigmentation is a reactive hypermelanosis secondary to cutaneous inflammation or wound healing and is more commonly observed in darker skin phototypes (Fitzpatrick IV–VI) because of the increased melanocyte activity and melanin production.^21^^,^^23^^,^^24^ In our study, patients had no endocrine disorders and did not use drugs that can induce pigmentation. Therefore, in the limited excision group, melanin production at the brown-colored area around the postoperative scar might have been promoted or regulated by factors such as sun exposure, inflammation, skin phototype, sex hormones, and aging, resulting in the accumulation of more melanin pigment and further hyperpigmentation.

Eliminating the causes of hyperpigmentation is necessary to achieve inconspicuous postoperative scars, thus highlighting the importance of postoperative care. In transverse facial cleft, because the patients are infants, scars around the oral commissure are more susceptible to movement, friction, and UV exposure, increasing the risk of wide scars and postinflammatory hyperpigmentation. Taping facilitates approximation of wound edges, reduces tension, decreases the risk of hypertrophic scarring, and protects against UV exposure, thereby mitigating these risks.^25^^,^^26^ Taping is typically performed approximately 12 weeks following suture removal.^26^ Moreover, because postoperative melanocyte activity varies by skin phototype, future studies should standardize postoperative care and stratify patients by skin phototype to evaluate whether complete excision of the pigmented white lip at the cleft margin differentially affects postoperative scarring. Analyses within the same skin phototype can clarify the effect of extended excision on periscar pigmentation, whereas comparisons across different phototypes can elucidate the effects of different skin phototypes on pigmentation.

The findings of this study indicate that the residual melanin pigment at the cleft margin contributes to the initial development of brown-colored areas around the postoperative buccal scar as well as their progressive darkening and increased visibility during growth. Additionally, when the excision area of the cleft tissue is minimized to reduce scarring, it may paradoxically result in more conspicuous scars due to pigment accumulation and deepening of the color intensity over time.

Several techniques, including Z-plasty,^7^ W-plasty,^11^ and straight-line closure,^12^ have been used for the repair of transverse facial cleft; however, to the best of our knowledge, the relationship between surgical techniques and the degree of postoperative periscar pigmentation has not been previously reported. In the present study, Z-plasty was used in all cases; therefore, differences in pigmentation outcomes among different techniques could not be determined. Additionally, there are numerous design variations within the same technique depending on the institution or the surgeon, making it difficult to evaluate how differences in surgical technique influence periscar pigmentation outcomes. However, based on our finding that excision of the pigmented white lip at the cleft margin can prevent postoperative periscar hyperpigmentation, we believe that minor adjustments to the surgical design with this principle in mind can lead to improved esthetic outcomes.

Extension of a transverse facial cleft beyond the anterior edge of the masseter muscle is uncommon.^4^ In the present study, the cleft length in the extended excision group was 8.9 ± 3.3 mm (range, 5–15 mm), and excision of the entire pigmentated white lip at the cleft margin did not increase scar tension; the reconstructed commissure exhibited favorable anatomy and function. This indicates that the cleft length is relatively short in most transverse facial clefts and that pigmentation excision may not considerably affect scar tension. However, because all participants in this study were Japanese, the area of conspicuous pigmentation might be wider in other ethnic groups, necessitating more extensive skin excision, or the cleft itself may be broader, resulting in greater scar tension. In such cases, avoiding excessive scar tension must be prioritized over pigmentation reduction, and postoperative pigmentation should instead be managed with adjunctive therapies such as laser treatment.

Surgical repair of transverse facial clefts is expected to produce favorable outcomes in oral function and appearance. Achieving both objectives in a single operation is desirable and feasible. Favorable esthetic outcomes can be achieved by removing all macroscopic pigmented white lip at the cleft margin. Pigmentation at the cleft margin was observed to extend outward from the vermilion–cutaneous junction to the white lip, and the skin excision area should be marked accordingly. Therefore, we recommend the following surgical design. First, identify and mark the anatomic landmarks of the lip. Second, determine the new commissure points on the cleft side with reference to the noncleft side. Third, mark the outline of the cleft tissue excision area while accounting for pigmentation of the white lip. If the pigmented area is unclear, adjust the surgical lighting or manually pinch the cleft skin to reproduce the skin color tone at closure. Finally, the Z-plasty skin closure technique may be applied, although its use remains controversial. Skin pigmentation and distortion are visible when the pigmented furrow extending from the commissure to the auricle is originally present. In such cases, additional therapeutic procedures, such as augmentation or laser therapy, may be required.

This study has some limitations. First, because of the small sample size, the groups were unevenly distributed (nine vs. seven participants), which limited the statistical power and generalizability of the results. Second, the retrospective design prevented the control of potential confounders such as lighting conditions, scar care differences, scar assessment timing, and follow-up uniformity. Third, all patients were Japanese and were recruited from two hospitals located in a single region, restricting the generalizability of pigmentation-related findings and highlighting the need for validation across diverse ethnicities and skin phototypes. Fourth, histological examination of the cleft margin was not performed on all patients, preventing the quantitative correlation between melanin density and clinical scar outcomes. Fifth, long-term follow-up was not performed, limiting assessment of the durability of the esthetic outcomes. Therefore, these findings should be regarded as preliminary, and studies with larger cohorts, prospective designs, and comprehensive histological assessment are warranted.

## Conclusion

In transverse facial cleft repair, the entire macroscopically pigmented region of the white lip adjacent to the cleft margin should be excised during commissuroplasty whenever feasible. Incorporating this approach into the surgical design can achieve more favorable esthetic outcomes for several years.

## Funding

None.

## Ethical approval

This study was approved by the Ethics Committee of Shinshu University School of Medicine (approval number: 5846).

## Declaration of competing interest

None declared.
